# Epigenetics in Plant Response to Climate Change

**DOI:** 10.3390/biology14060631

**Published:** 2025-05-29

**Authors:** Wei Zhou, Min Wang, Lishan Wang, Yinghui Liu, Zaimin Tian, Linan Xie, Yu Wang

**Affiliations:** 1 College of Pharmacy, Hebei North University, Zhangjiakou 075000, China; 17608203186@163.com; 2 Hebei Provincial Key Laboratory of Neuropharmacology, Zhangjiakou 075000, China; 3 College of Agriculture and Forestry Science and Technology, Hebei North University, Zhangjiakou 075001, China; wangmin2451@163.com (M.W.); leely519@126.com (Y.L.); nkxtzm@163.com (Z.T.); 4 National Key Laboratory of Crop Genetic Improvement, National Engineering Research Center of Rapeseed, Hubei Hongshan Laboratory, Huazhong Agricultural University, Wuhan 430070, China; wanglishan5433@163.com; 5 School of Ecology, Northeast Forestry University, Harbin 150040, China; 6 Key Laboratory of Sustainable Forest Ecosystem Management-Ministry of Education, School of Ecology, Northeast Forestry University, Harbin 150040, China; 7 College of Life Science, Northeast Forestry University, Harbin 150040, China; 8 Key Laboratory of Saline-alkali Vegetation Ecology Restoration, Northeast Forestry University, Ministry of Education, Harbin 150040, China

**Keywords:** DNA methylation, histone modification, non-coding RNA, epigenetics, climate change

## Abstract

Since the last glacial maximum, the Earth has experienced drastic climate changes. However, with the advent of the Industrial Revolution, human activities have abruptly intensified, altering the planet’s climate patterns. Climate models predict that over the next hundred years, the globe will experience even more drastic changes than those observed in the past 20,000 years. Understanding how plants adapt to environmental changes has thus become a critical research priority. Currently, evidence suggests that plants adapt to environmental changes primarily through two distinct pathways: one mediated by genetic variation and the other involving the direct or indirect participation of epigenetic modifications. Given the unprecedented rate of contemporary climate change, epigenetic mechanisms may exert a more immediate and flexible influence than genetic modifications. Epigenetics, including DNA methylation, histone modification, and non-coding RNA, plays an important role in the post-transcriptional regulation process and affects the growth and development of plants, as well as their responses to environmental changes. In this review, we synthesize current knowledge on the epigenetic mechanisms underlying plant responses to environmental changes and examine the dynamic interplay between epigenetic regulation and environmental stimuli, aiming to provide insights into enhancing the tolerance of plants to environmental stress factors.

## Simple Summary

In this review, we meticulously summarize the intricate epigenetic mechanisms and fascinating phenomena underlying plants’ responses to diverse environmental changes, including abiotic stresses such as extreme temperatures, drought, salinity, and heavy metal exposure. By delving into the molecular processes of DNA methylation, histone modification, and non-coding RNA-mediated regulation, we aim to elucidate how these epigenetic modifications orchestrate plants’ physiological, biochemical, and developmental adjustments in response to environmental cues. Furthermore, we systematically explore the dynamic and bidirectional interactions between plant epigenetics and environmental changes. This involves not only understanding how environmental stimuli trigger epigenetic alterations but also investigating how these epigenetic changes, in turn, influence plants’ perception, adaptation, and short-term and long-term memory of environmental stressors. We summarize the progress made in recent years, aiming to provide insights into enhancing the tolerance of plants to environmental stress factors.

## 1. Introduction: Climate Change, Plant Adaptation Challenges, and the Epigenetic Dimension

Climate change represents a formidable challenge to global ecosystems, characterized by unprecedented rates of warming and alterations in weather patterns [1]. This escalating challenge manifests as an increase in the intensity, duration, and frequency of abiotic stressing events, including drought, heat waves, extreme temperatures, fluctuating water availability, soil salinity, nutrient deficiencies, and altered CO_2_ and light intensity levels [2,3]. These abiotic factors, alongside associated biotic challenges such as shifts in pathogen and pest recurrence, severely impact plants’ growth, survival, biodiversity, and ecosystem stability. To cope with these dynamic and increasingly severe environmental pressures, plants have evolved intricate mechanisms to perceive signals and adjust their gene expression and metabolism [4,5]. Beyond the classical genetic basis of inheritance, mounting evidence highlights epigenetics as a crucial regulatory layer enabling adaptation strategies. How climate change affects soil pollution and the mobility of heavy metal ions in soil is a relatively understudied research area. This long-term environmental change may influence the transport of heavy metal ions related to pollution, such as mercury, lead, and chromium, thereby increasing organisms’ exposure to these contaminants. For instance, the accelerated melting of Arctic glaciers may enhance the mobility of lead [6]. In soils with low pH values, metal ions are more easily leached. Excessive rainfall may lead to soil acidification, which increases metal ions’ solubility and bioavailability for plant uptake. Numerous studies have shown that lead-contaminated urban soils tend to become suspended when evapotranspiration is highest and soils are driest during summer and autumn. These particles can settle in other areas, potentially contaminating clean soils [7]. Additionally, higher soil temperatures are associated with higher metal concentrations in crops [8].

Plant epigenetics encompasses a complex network of molecular mechanisms that regulate gene expression without altering the DNA sequence [9]. The core mechanisms of epigenetic regulation in plants include DNA methylation, histone modifications, and non-coding RNAs. These modifications dynamically modulate chromatin structure and the accessibility of transcriptional machinery, thereby controlling gene expression and enabling rapid reprogramming in response to environmental stimuli [10]. For sessile organisms like plants, which cannot escape adverse conditions, the ability to rapidly adjust their physiology and development is paramount for survival [11]. Traditional genetic approaches, relying on spontaneous mutations and the relatively slow process of natural selection, are often insufficient to keep pace with the accelerated rate of anthropogenic climate change [12,13]. Epigenetic mechanisms provide a flexible and responsive alternative by allowing rapid and reversible changes in gene expression without altering the DNA sequence, enabling plants to acclimatize to environmental fluctuations within an individual’s lifespan [14]. Furthermore, stress-induced epigenetic changes can be maintained through mitotic divisions and, in some cases, transmitted transgenerationally, providing a mechanism for stress memory or priming that can enhance the response of subsequent generations to recurring challenges [15,16,17]. Understanding the molecular mechanisms underlying plant responses to climate change, particularly the role of epigenetic regulation, has become a major research objective. This review aims to summarize current knowledge on how epigenetic mechanisms contribute to plants’ adaptation and resilience in the face of climate change. By elucidating these mechanisms, researchers can identify novel strategies for enhancing stress tolerance and developing climate-resilient plants.

## 2. Core Epigenetic Mechanism

### 2.1. DNA Methylation

**Figure 1:**
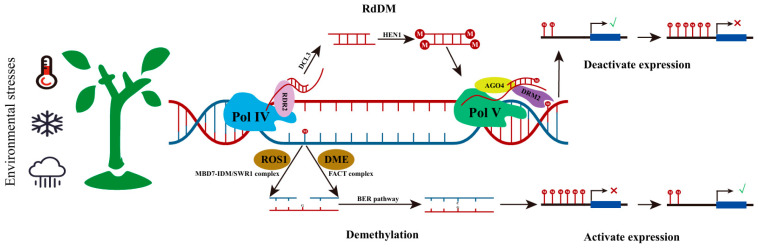
DNA methylation in response to environmental stress. When plants perceive external environmental changes (such as high temperatures, freezing, extreme precipitation, etc.), they adapt to such environmental stresses by modulating the methylation levels of related genes. Changes in methylation levels in promoter regions are typically associated with gene expression, where the addition or removal of methylation in promoter regions can inhibit or activate the expression of corresponding genes. The addition or removal of methylation is primarily driven by two opposing processes: de novo methylation mediated by the RdDM (RNA-directed DNA methylation) pathway and active demethylation promoted by ROS1 (Repressor of Silencing 1) and DME (DEMETER).

DNA methylation is one of the most extensively studied epigenetic modifications in plants. In eukaryotes, DNA methylation requires the participation of S-adenosyl methionine (SAM) and DNA methyltransferases. This process leads to the addition of a methyl group from SAM to the fifth carbon of cytosine, while the underlying DNA sequence remains unchanged and is stably inherited during cell division [18,19]. Plant DNA methylation occurs in three sequence contexts: CG, CHG, and CHH (where H represents A, T, or C). In *Arabidopsis thaliana*, the CG methylation rate is the highest, accounting for 24% of the whole-genome methylation level; CHG methylation constitutes 6.7%, while the CHH methylation level is the lowest (1.7%), mainly existing in transposons and other repetitive sequences [20,21]. Plant DNA methylation involves three processes: de novo synthesis, maintenance, and demethylation. De novo methylation refers to the process of adding methyl groups to unmethylated DNA sequences, which is mainly achieved through the RNA-directed DNA methylation (RdDM) pathway. The canonical RdDM is that SAWADEE HOMEODOMAIN HOMOLOGUE 1 (SHH1) recognizes H3K9me2 and interacts with SNF2 DOMAIN-CONTAINING PROTEIN CLASSY1 (CLSY), recruiting RNA POLYMERASE IV (Pol IV) to the RdDM locus [22,23] and then transcribing the relevant sequences to form single-stranded RNA. The Pol IV-derived ssRNA is then converted into double-stranded RNA (dsRNA) by RNA-DEPENDENT RNA POLYMERASE 2 (RDR2) [24,25], which is subsequently cleaved by DICER-LIKE PROTEIN 3 (DCL3) to generate 24 nt small interfering RNAs (siRNAs) [26]. This 24 nt siRNA is finally loaded onto ARGONAUTE 4 (AGO4) [27,28,29]. Subsequently, scaffold RNAs produced by RNA POLYMERASE V (Pol V) transcription form complementary pairs with the 24 nt siRNAs [30], recruiting DOMAINS REARRANGED METHYLTRANSFERASE 2 (DRM2) to catalyze de novo DNA methylation [31,32]. After the establishment of de novo methylation, the maintenance of diverse DNA methylation patterns in plants requires an expanded repertoire of methyltransferases. The maintenance of CG methylation in plants shares mechanistic similarities with mammals, owing to the homology between the plant CG maintenance methyltransferase METHYLTRANSFERASE 1 (MET1) and its mammalian counterpart DNA METHYLTRANSFERASE 1 (DNMT1). Following DNA replication, hemi-methylated CG dinucleotides are generated, which are specifically recognized by MET1. This enzyme then methylates the unmodified cytosine on the newly synthesized DNA strand, thereby faithfully maintaining the parental methylation pattern [33,34]. The maintenance of CHG methylation is regulated by CHROMOMETHYLASE 3 (CMT3) and CHROMOMETHYLASE 2 (CMT2) [35,36]. When recruited by H3K9me2, CMT3 can directly add methylation on target sequences, while also recognizing CHG methylation marks through the histone methyltransferase KRYPTONITE (KYP), catalyzing histone methylation to reinforce the silencing in these regions [37]. In *Arabidopsis*, an impaired histone demethylase function enhances the association between H3K9me2 and CMT3, leading to elevated CHG methylation at specific loci [38]. Conversely, mutations in *Arabidopsis* H3K9-specific methyltransferases result in significantly reduced CHG methylation levels [39]. The methylation of CHH is maintained by CMT2 and DRM2, according to the genomic location [40]. CMT2 is targeted and recruited by H3K9me2, primarily maintaining CHH methylation in pericentromeric heterochromatin [37], while DRM2 is responsible for CHH methylation at the borders of long transposable elements in heterochromatin and short transposable elements in euchromatin [35,41,42]. The active DNA demethylation in plants is mainly achieved by REPRESSOR OF SILENCING 1 (ROS1) and DEMETER (DME) recognizing the methylated cytosine and through base excision repair [43,44]. In plants, DNA methylation plays a crucial role in various biological processes, including gene regulation, transposable element silencing, and genomic imprinting. Under environmental stress, dynamic changes in DNA methylation (either gain or loss) can lead to gene silencing or activation. This plasticity enables plants to rapidly adapt their gene expression patterns in response to environmental stimuli [45] (Figure 1).

N6-methyldeoxyadenosine (6mA) is a recently discovered DNA modification that is involved in regulating the adaptation of plants to abiotic stress. Analysis of 6mA’s distribution across different genomic regions (5′UTR, exon, intron, 3′UTR) and gene types (protein-coding genes, miRNAs, snoRNAs, etc.) shows that 6mA is more abundant in gene body regions, primarily enriched in exons, and most 6mA-modified genes are protein-coding genes. 6mA is associated with actively expressed genes in Arabidopsis and regulates genes involved in important biological pathways, potentially participating in plant vegetative growth, photosynthesis, and stress adaptation [46].

### 2.2. Histone Modification

**Figure 2:**
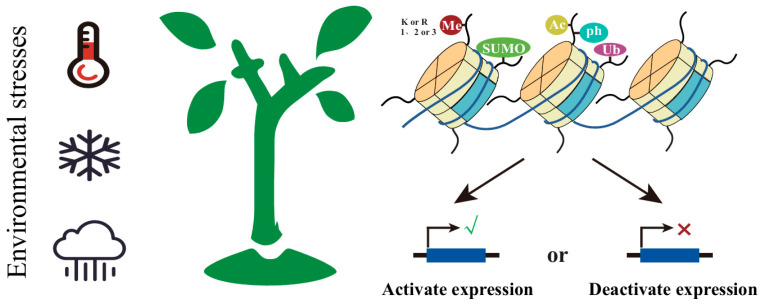
Plants respond to environmental stresses by regulating the expression levels of stress-related genes through changes in the levels of different histone modifications. These dynamic modifications of histones, including methylation, SUMOylation, acetylation, and ubiquitination, can alter chromatin structure and accessibility, thereby influencing the transcriptional activity of target genes. Me, methylation; K, lysine; R, arginine; 1, monomethylation; 2, Dimethylation; 3, Trimethylation; SUMO, SUMOylation; AC, acetylation; Ub, ubiquitination.

Eukaryotic nucleosomes represent the fundamental unit of chromatin, consisting of two structural components: (1) a histone octamer core comprising two copies each of H2A, H2B, H3, and H4 and (2) approximately 147 bp of DNA wrapped in 1.75 superhelical turns around this octamer. Both of these constitute the basic morphology of nucleosomes [47]. Histone modifications mainly come from the modifications of various amino acids on the N-terminal to C-terminal of the tails of each histone subunit, which can form a variety of histone modifications including methylation, acetylation, ubiquitination, etc. These modifications serve as crucial epigenetic marks that regulate chromatin accessibility, influencing the binding of polymerases and transcription factors to modulate gene expression [48] (Figure 2). Histone methylation depends on its position and state and is related to either repression or activation. For example, the formation of H3K27me3 methylation is mainly through PRC2 (Polycomb Repressive complexes 2) [49]. Histone demethylation mainly includes two evolutionarily conserved families: Lys-specific demethylase (LSD) demethylases and Jumonji C (JMJC) demethylases. Both LSD1 and LSD2 catalyze flavin adenine dinucleotide (FAD)-dependent amino-oxidation reactions to demethylate the substrates [50]. All histone modifications follow a set of “read–writer–eraser” establishment and removal processes. There are a total of 21 JMJC homologous proteins in *Arabidopsis thaliana*, which are involved in different histone demethylation activities in the genome [51]. The four FAD-dependent lysine-specific histone demethylases in *Arabidopsis thaliana* (LDL1, LDL2, LDL3, and FLD) are all involved in the regulation of flowering time [52].

Histone acetylation is mainly related to gene activation, mainly occurs at the relatively conserved lysine positions in the N-terminal of H3 and H4, and is coordinated by histone acetyltransferases and histone deacetylases [53]. In *Arabidopsis thaliana*, SAP AND MIZ1 DOMAIN-CONTAINING LIGASE1 (SIZ1) inhibits the binding of histone deacetylase 6 (HDA6) to its target genes *FLOWERING LOCUS C* (*FLC*) and *MADS AFFECTING FLOWERING 4* (*MAF4*), leading to increased histone H3 acetylation levels and a consequent upregulation of *FLC* and *MAF4* expression [54]. OTLD1 is a member of the ovarian tumor (OTU) deubiquitinase family that deubiquitinates histone H2B and regulates growth-related genes. Through *Arabidopsis thaliana* gain-of-function and loss-of-function mutant lines of OTLD1, its dual regulatory roles in both transcriptional repression and activation were revealed. This transcriptional activation activity of OTLD1 involves (1) its occupancy on target chromatin, (2) the deubiquitination of monoubiquitinated H2B within the occupied regions, and (3) the establishment of euchromatic histone acetylation and methylation marks [55]. AtAurora1 is an *Arabidopsis thaliana* kinase that mediates the cell cycle-dependent phosphorylation of histone H3 at serine 10 (H3S10ph). In plants, this phosphorylation exhibits crosstalk with adjacent histone modifications, including the methylation, acetylation, or phosphorylation of neighboring amino acid residues [56].

### 2.3. Non-Coding RNA

**Figure 3:**
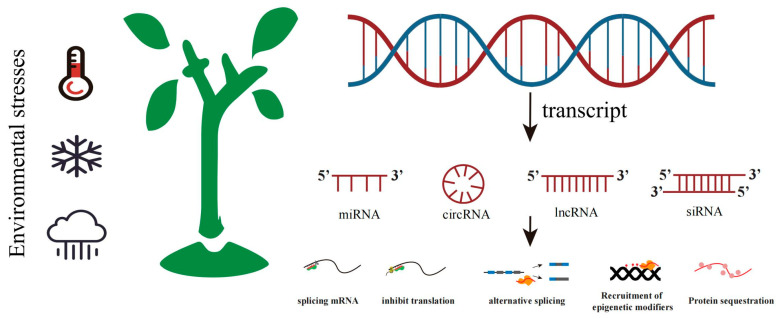
Non-coding RNAs in plants respond to environmental stress. When plants are subjected to external environmental stress, they will generate various non-coding RNAs, including microRNAs (miRNAs), circular RNAs (circRNAs), long non-coding RNAs (lncRNAs), and small interfering RNAs (siRNAs). Plants regulate stress-responsive genes through translational, transcriptional, and epigenetic modifications to adapt to environmental challenges.

Non-coding RNAs are widely present in various organisms. With the continuous im-provement in high-throughput sequencing technologies, a large number of non-coding RNAs have been identified, and their functions and mechanisms of action have gradually been elucidated. Non-coding RNAs regulate the processes of transcription and translation in various ways, thereby influencing plants’ responses to climate change (Figure 3). Based on their structural configurations and molecular states, ncRNAs can be categorized into linear ncRNAs and circular RNAs (circRNAs). Linear ncRNAs are further subdivided into long non-coding RNAs (lncRNAs) and small non-coding RNAs, with the latter group including miRNAs (microRNAs) and siRNAs. CircRNAs are primarily formed through back-splicing, wherein the downstream 5′ splice donor site joins the upstream 3′ splice acceptor site. Lacking a 5′ methylated cap and 3′ poly (A) tail, circRNAs exhibit high stability due to their resistance to RNase degradation [57,58]. Notably, studies have found that circRNA has the potential for protein coding and exerts biological functions by encoding polypeptides [59]. LncRNAs, typically greater than 200 nt in length, may originate from intergenic regions, exonic sequences, or intronic regions. In plants, lncRNAs are usually transcribed by RNA polymerase II (Pol II); however, pol III, pol IV, and pol V can also participate in lncRNA transcription [60]. Usually, the expression of lncRNA is tissue-specific, and many lncRNAs are regulated during specific developmental processes and environmental changes. Plant miRNAs are typically 20–24 nucleotides (nt) in length. Their biogenesis involves four key steps: (1) transcriptional initiation, (2) precursor processing, (3) methylation modification, and (4) RNA-induced silencing complex (RISC) assembly. miRNA genes are first transcribed by RNA polymerase II (Pol II) to generate primary miRNA transcripts (pri-miRNAs). Then, through the action of DICER-LIKE proteins, double-stranded miRNAs are processed from pri-miRNAs. The 3′ end of the double-stranded miRNA undergoes methylation modification catalyzed by the HEN1 methyltransferase, yielding mature miRNAs. Finally, one strand of the mature miRNA is further loaded onto the ARGONAUTE (AGO) protein, and eventually, miRNA-induced silencing complex (miRISC) is formed. This complex mediates post-transcriptional gene silencing through perfect or near-perfect complementary base pairing with target mRNA [61]. In general, siRNAs are typically 21–24 nt in length and originate from double-stranded RNAs (dsRNAs), and they are generated by the cleavage of DCL2/DCL3/DCL4. siRNAs are mainly classified according to their sources as follows: (1) trans-acting siRNAs (ta-siRNAs) that originate from long single-stranded RNAs, (2) heterochromatic siRNAs (hc-siRNAs) that are derived from intergenic/repetitive genomic regions, (3) siRNAs derived from transposable elements (TEs) that belong to repetitive DNAs or transposons, (4) repeat-associated siRNAs (ra-siRNAs), and (5) long small interfering siRNAs (lsiRNAs). In addition to these, there is another class of secondary siRNAs called phased small interfering RNAs (phasiRNAs), which are usually produced from PHAS loci. Plant siRNAs need to be completely matched with target genes to exert their functions [62].

## 3. The Role of Epigenetic Modification in Plants’ Adaptation to Climate Changes

### 3.1. The Role of DNA Methylation in Plants’ Adaptation to Climate Changes

Plants are highly susceptible to environmental fluctuations during growth, leading to the evolution and development of a series of protective mechanisms. DNA methylation plays an important role in this process. Drought can cause changes in the DNA methylation of rice, and these changes will be retained after the drought is relieved [63]. Under water stress treatment, drought-tolerant maize inbred lines exhibit significantly elevated DNA methylation levels, compared to drought-sensitive lines [64]. Populus tomentosa under different geographical conditions shows differential epigenetic regulation under drought conditions. The non-CG methylation level in the promoter region of plants in the south is lower than that of plants at high latitudes, indicating a lower drought tolerance ability [65]. In barley, water deficiency induces numerous differentially methylated sites (DMSs) in leaves and roots; most of the differentially methylated sites are reversible after rewatering, indicating that DNA methylation serves as a crucial adaptive response to water stress [66]. The methylation level in the promoter region of *glyceraldehyde-3-phosphate dehydrogenase, cytosolic* (*TaGAPC1*) in wheat is induced by osmotic stress and salt stress, leading to expression level changes [67]. The formation and maintenance of the drought stress memory in *Medicago ruthenica* are closely associated with DNA methylation. After drought stress, the genome-wide methylation level decreases [68]. Differential methylation patterns regulate the selective expression of energy metabolism-related genes, enhancing the metabolism of *Medicago sativa* L. after drought stress [69]. *Brassica napus* responds to polyethylene glycol (PEG) treatment by upregulating the methylation levels in the promoter region, exon 1, exon 2, and intron 1 region of the *Delta 1-Pyrroline-5-Carboxylate Synthetases* (*BnP5CSA*) gene [70].

Many studies indicate that climate change affects the leaching potential and bioavailability of heavy metal ions in soils [71,72]. Plants absorb heavy metals mainly in two ways: root absorption and leaf absorption [73]. Exposure to an environment with excessive heavy metal ions can induce toxicity, triggering a series of epigenetic modification to counteract these toxic effects. The *Colorless nonripening* (Cnr) mutant of tomato regulates the expression of *PECTIN METHYLESTERASE53* (*SlPME53*) through METHYLTRANSFERASE 1 (SlMET 1)-mediated CG hypermethylation, thus regulating the content of apoplastic iron [74]. Under Mn/Cd stress, pokeweed shows increasing numbers of differentially methylated sites with rising stress concentrations [75]. Under Cd stress, hybrid kenaf seedlings show significant Cd resistance advantages in terms of morphology and antioxidant enzyme activities. Through methylation-sensitive amplified polymorphism (MSAP) analysis, it is found that the total DNA methylation level under cadmium stress decreases by 16.9% in F1 and increases by 14.0% and 3.0% in the parents, respectively. Principal coordinate analysis (PCoA) shows that there is a significant epigenetic differentiation between F1 and the parents under cadmium stress. Virus-induced gene silencing (VIGS) knockdown of the differentially methylated gene NPF2.7 increases the sensitivity of kenaf seedlings to cadmium stress [76]. In an *Arabidopsis* population, Cd treatment resulted in higher levels of CpG DNA methylation compared to controls, accompanied by the upregulation of genes involved in symmetric methylation and histone deacetylation [77]. Under Pb, Cd, and Zn metal stress, CG DNA hypomethylation is found in the promoter region of the metal detoxification transporter in wheat, which indicates that DNA methylation regulates the expression of the metal detoxification transporter to confer metal toxicity resistance [78]. In rice, the *METHYLTRANSFERASE 1* (*OsMET1-2*) heterozygous mutants of a mercury-resistant line demonstrated significantly enhanced survival rates, improved oxidative stress tolerance, and reduced mercury uptake/transport compared to wild-type plants following Hg treatment; the upregulation of mercury resistance-related genes in the *OsMET1-2* heterozygous mutant is highly correlated with the DNA hypomethylation in their promoter regions [79]. Furthermore, the Microrchidia6 (OsMORC6) protein positively regulates the expression of genes related to the plant cell wall and oxidative stress under Cd stress by mediating DNA methylation [80]. By studying the responses of 8 Amaranthus, 5-MTase genes and 2 DMTase genes to heavy metals (Cd, Pb, Zn, Mn) and their combinations (Cd/Pb, Cd/Zn, Pb/Zn) in root and leaf tissues, it is shown that the Amaranthus MTase and DMTase genes participate in heavy metal stress responses by regulating DNA methylation and demethylation [81]. The phytotoxicity of Cd leads to a reduction in the stem biomass of *Zataria multiflora*, while Nitric oxide (NO) and ascorbate (Asc) can alleviate this toxicity. This phenomenon was associated with extensive hypomethylation, which conferred Cd tolerance through the transcriptional upregulation of terpenoid and phenylpropanoid metabolism, increased proline concentrations, and improved antioxidant capacity [82].

Soil salinization severely affects plants’ growth; high concentrations of Na^+^ in the soil cause salt stress, disrupt the plant’s water circulation system, lead to toxic ion accumulation, and ultimately result in plant toxicity. Currently, the mechanisms by which DNA methylation mediates plants’ salt tolerance remain unclear and exhibit species-specific variations. Following salt stress induction, the activity of total DNA methyltransferase in soybean seedlings decreases, leading to DNA hypomethylation [83]. In contrast, a high salt treatment in *Medicago sativa* will lead to a significant increase in the whole-genome methylation level [84]. In sugar beet, salt induces substantial changes in methylation patterns, with hypermethylation occurring more frequently than hypomethylation in *Beta vulgaris* ssp. *maritima* [85]. For *KOW DOMAIN-CONTAINING TRANSCRIPTION FACTOR1* (*KTF1*), an essential component of the RdDM pathway, the maize salt-sensitive mutant *ZmKTF1* exhibits significantly reduced genome-wide CHH methylation. Under salt stress, ZmKTF1-mediated DNA methylation regulates the expression of multiple redox enzyme genes, while increasing reactive oxygen species (ROS) levels [86]. Research demonstrates that the hypomethylation of key genes such as 1-amino cyclopropane-1-carboxylic oxidase 1 (*ACO1*) in citrus, coupled with a higher chromatin accessibility, activates stress-responsive pathways and enhances salt tolerance in tetraploid plants [87]. Strigolactones can significantly upregulate the key genes related to the phosphatidylinositol signaling system in tomatoes under salt stress and reduce the CHG methylation levels of the promoters and gene body regions to promote their expression [88]. After treatment with 5-azadC, the expression of the DNA methyltransferase gene in tomatoes is upregulated, and the expressions of *DEMETER-LIKE 1* (*SlDML1*), *DEMETER-LIKE 3* (*SlDML3*), *DEMETER-LIKE 4* (*SlDML4*), *High-affinity K^+^ transporter 1* (*SlHKT1*), *Na^+^/H^+^ antiporter 1* (*SlNHX1*), and *Salt Overly Sensitive 1* (*SlSOS1*) are downregulated to alleviate salt stress [89].

Extreme temperatures represent another major factor affecting plant survival. For example, DNA methylation in cucumbers is related to the radicle elongation rate under low-temperature stress, and it may be partially regulated through dynamic changes in the methylation pattern [90]. Rice *Nucleotide-binding leucine-rich repeat receptors* (*NLR*) genes exhibit significant methylation at the CG sites, and many rice *NLR* genes show changes in DNA methylation after low-temperature stress [91]. Through the transcriptomes analysis of birch in two habitats with different temperature ranges, it was revealed that the lignin biosynthesis-related genes and transcription factors show a significantly reduced expression at higher temperature ranges. Whole-genome bisulfite sequencing showed that the DNA methylation in two NAC-domain TFs-NAC secondary wall thickening promoting factor1/2 (*BpNST1/2*) and secondary wall-associated NAC domain protein1 (*BpSND1*) promoter may be involved in inhibiting the expression of these genes, thus reducing lignin content [92]. Using the rapid clonal propagation of *Lemna minor*, researchers found that a high temperature induces DNA hypermethylation under many CG and CHG cytosine contexts, rather than CHH. In addition, even after being cultured for 3 to 12 generations in a normal environment, the differential methylation observed in the CHG context can still be detected [93]. Compared with controls, a high temperature will induce DNA demethylation in barley, while mild low temperatures predominantly induce DNA methylation [94].

In lotus, *Arabidopsis*, and rice, 6mA is primarily enriched around transcription start sites, showing a positive correlation with gene expression levels, and it preferentially accumulates in longer genes with more exons [95]. Studies demonstrate that under both normal and cold conditions, 6mA peaks predominantly localize in gene body regions. Genome-wide 6mA levels increase in *Arabidopsis* and rice after cold treatment, with methylated-up genes significantly enriched in various biological processes, while no significant enrichment is observed in methylated-down genes. Correlation analyses reveal a positive relationship between 6mA levels and gene expression [96]. The 6mA modification is dynamically regulated by adenine methyltransferases (e.g., METTL) and demethylases (e.g., ALKBH). In the rice genome, 20% of genes and 14% of transposable elements carry 6mA modifications, with primary enrichment in intergenic regions. Research indicates that ALKBH1 can remove 6mA from R-loops in rice, inhibiting H3K27me3 by antagonizing the binding of PRC2 to target genes, thereby promoting gene expression [97,98]. Rice 6mA levels significantly decrease under cold stress but increase under heat or salt stress. These levels negatively correlate with cold tolerance but positively correlate with salt and heat tolerance. Mutations in DDM1 lead to growth defects and reduced 6mA levels in plants [99].

### 3.2. The Role of Histone Modification in Plants’ Adaptation to Climate Changes

Histone methylation, one of the important histone modifications, serves as a major determinant for the formation of genomic structure, as well as the activated and silenced regions of the genome, playing an indispensable role in plants’ response to environmental changes. Under drought stress, the chromatin interaction between the histone demethylase Jumonji 17 (JMJ17) and protein kinase OPEN STOMATA 1 (OST1) in *Arabidopsis thaliana* is attenuated, leading to decreased H3K4me3 modification levels at the *OST1* locus and consequent upregulation of its expression [100]. Nguyen et al. [101] used mannitol to simulate osmotic stress and found that the stress induced an increased level of H3K4me3 (an active mark) in both the promoter region and the transcription start site (TSS) region of *AtMYB44*, while increased H3K4me3 modifications were detected in downstream targets *ABA INSENSITIVE 1* (*ABI1*), *ABA INSENSITIVE 2* (*ABI2*), and *HIGHLY ABA-INDUCED PP2C GENE 1* (*HAI1*) [102]. Further evidence demonstrates that the H3K9 demethylase JMJ27 modulates histone methylation levels of drought-responsive genes via its demethylase activity, thereby positively regulating drought stress responses [103]. In rice, the histone demethylase JMJ710 targets the chromatin of the *MYB48-1* gene, catalyzing H3K36me2 demethylation to suppress *MYB48-1* expression. Under drought conditions, the downregulation of JMJ710 relieves this repression, activating *MYB48-1* and its downstream drought-responsive genes to confer the tolerance [104]. Zong et al. [105] reported that drought stress induces the upregulation of four rice dehydrin genes, accompanied by a significant increase in H3K4me3 (an activation mark) and decrease in H3K27me3 (a repressive mark) at their genomic loci. Additionally, in tomatoes, drought stress induces a widespread reduction in the repressive mark H3K9me2 across all regions of *ABA/water stress/ripening-induced* (*Asr2*) [106], while the upregulation of *ABA/water stress/ripening-induced* (*Asr1*) expression is closely related to an decrease in the level of the repression marker H3K27me3 [107]. Sani et al. [108] demonstrated that Na^+^ pretreated *Arabidopsis thaliana* exhibited enhanced drought tolerance compared to untreated controls. This response was associated with marked epigenetic modifications in *High-affinity K^+^transporter 1* (*HKT1*), which encodes a high-affinity Na^+^ transporter. Reduced H3K27me3 enrichment at the *HKT1* locus facilitated a rapid yet transient increase in *HKT1* mRNA levels, mechanistically explaining the physiological effects of this priming treatment. The rice H3K4 methyltransferase SET DOMAIN GROUP (OsSDG721) binds to the promoter and coding region of *OsHKT1;5* and increases the H3K4me3 marker, thus upregulating the expression of *OsHKT1;5* under saline-alkali stress [109]. Under high salt stress, the rapid decrease in the modification level of H3K27me3 in the promoter region of *OsMYB91* mediates the expression of this gene [110]. In soybean seedlings under salt stress, genes associated with hypomethylated DNA regions also showed higher average levels of the active histone marker H3K4me3 and lower average levels of the repressive histone marker H3K4me2; transcriptomic analysis supports the role of DNA hypomethylation in fine-tuning the chromatin state to enhance gene expression [83]. In *Arabidopsis*, *AT-hook motif nuclear localized protein 10* (*AHL10*) was found to recruit the SU(VAR)3-9 homologs (SUVH2/9) to the AT-rich DNA sequences in the nuclear matrix attachment regions (MARs) of the promoters of salt-responsive genes, promoting H3K9me2 deposition and inhibiting salt-responsive genes [111].

During rice seed development, a high temperature is closely related to the decrease in the expression levels of *MADS-box genes* (*OsMADS82*), *MADS-box genes* (*OsMADS87*), and *AGAMOUS-LIKE36* (*AGL36*) and the increased level of the repression marker H3K27me3 in their gene regions [112]. Compared to mild low temperatures, barley under high-temperature conditions exhibits more differentially expressed genes with higher levels of H3K9ac and H3K4me3, which may lead to chromatin decondensation and subsequent gene activation [94]. In wheat, H3K36me3 levels at the key vernalization gene *VERNALIZATION1* (*VRN1*) remain high after cold exposure during vernalization, maintaining its active state; in the absence of H3K36me3, *VRN1* expression significantly decreases after vernalization cold exposure memory [113]. Under low-temperature stress, the deposition of H3K27me3 markers in the gene body region of the *AGAMOUS* (*RcAG*) gene in China rose (*Rosa chinensis*) inhibits the transcription level of *RcAG*, resulting in the production of more petals [114].

Histone acetylation modifications are typically found in transcriptionally active chromatin regions, while the deacetylation of chromatin regions is considered to repress the associated gene expression [115]. In *Arabidopsis*, the E3 SUMO ligase HIGH PLOIDY2 (HPY2) catalyzes the SUMOylation of ARABIDOPSIS RESPONSE REGULATORs (ARR1) at K236, enhancing histone H3 acetylation and leading to transcriptional activation of ARR1 under cold and cytokinin conditions [116]. The rice histone deacetylase (HDAC) OsHDA716 mediates the deacetylation of OsbZIP46, reducing its DNA-binding capacity and transcriptional activity, thereby decreasing OsbZIP46 protein stability and suppressing cold tolerance in rice [117]. Under heat stress, the *Arabidopsis* zinc-finger protein SOMNUS (SOM) and histone mark reader EARLY BOLTING IN SHORT DAY (EBS) form a complex. SOM expression is activated by the AGL67-EBS complex through histone H4K5 acetylation, ultimately inhibiting seed germination [118]. Under high-temperature stress, the H3K9ac modification levels in promoter regions of maize heat shock transcription factor genes *Heat stress factor* (*ZmHsf-01*) and *ZmHsf-15* are significantly increased [119], H3K9ac and H4K5ac also show positive correlations with the expression of lateral root development-related genes *Haem Oxygenase-1* (*HO-1*) and *Giberellic Acid-Stimulated Like-1* (*GSL-1*) [120]. Heat stress in *Arabidopsis* causes heterochromatin disruption accompanied by histone hyperacetylation and DNA hypomethylation. Two plant-specific histone deacetylases, histone deacetylases (HD2B) and HD2C, can promote DNA methylation and alleviate heat-induced heterochromatin decondensation [121]. Chromatin Immunoprecipitation (ChIP) analysis under heat stress shows that histone acetyltransferase GCN5 is enriched at the *Heat Stress Factor* (*HSFA3*) and *UV-HYPERSENSITIVE 6* (*UVH6*) promoter regions, catalyzing the acetylation of H3K9 and H3K14 in these regions, thereby inducing HSFA3 and UVH6 expression [122]. Additionally, heat stress promotes increased H3K56ac levels in the exon regions of *HEAT SHOCK FACTOR* (*HsfA2*) and *HEAT SHOCK PROTEIN genes* (*Hsa32*), participating in the activation of HsfA2 and Hsa32 expression [123]. The histone methylation readers MRG1 and MRG2 (morf-related gene 1 and 2) promote hypocotyl elongation under warm environmental conditions. Transcriptome sequencing analysis indicates that MRG1/2 and phytochrome interacting factor 4 (PIF4) co-activate heat-responsive genes [124].

Zheng et al. [125] found that under high salt, histone acetyltransferase General control non-repressed protein 5 (GCN5)-mediated H3K9ac and H3K14ac modifications participate in activating the expression of *chitinase-like gene* (*CTL-1*), *polygalacturonase involved in expansion-3* (*PGX3*), and *MYB domain protein-54* (*MYB54*). When the roots of hydroponically cultured maize were stimulated with high salt, the mRNA expression levels of two *Histone acetyltransferase* (*HAT*) genes (*ZmHATB* and *ZmGCN5*) increased, leading to elevated global H3K9 and H4K5 acetylation levels and the transcriptional upregulation of expansins and other cell wall-related genes. Among these genes, both *β-expansin* (*ZmEXPB2*) and *xyloglucan endotransglucosylase* (*ZmXET1*) exhibited increased H3K9 acetylation levels in their promoter regions [126]. In sugar beet, the high expression of *peroxidase* (*POX*) is also accompanied by an increase in the acetylation of H3K9 and H3K27 in the coding region when under salt stress [127]. The rice histone H4 deacetylase Histone deacetylase (OsHDA706) regulates the expression of *2C type protein phosphatases* (*OsPP2C49*) through the deacetylation of H4K5 and H4K8, participating in the response to salt stress [128]. In Oryza species, the tissue-specific histone H4 variant (H4.V) enhances salt tolerance through mediating specific epigenetic changes. H4.V is specifically incorporated into heterochromatic sites, blocking the deposition of active histone marks. Salt stress triggers the redistribution of H4.V, allowing the acetylated histone H4K5ac to be integrated into the gene body region. An abnormal expression of H4.V will lead to defects in reproductive development and an impaired salt stress response [129].

Drought and heavy metal stress are also significantly impact plant survival. Under drought stress, ChIP analysis in sea buckthorn showed that 6 genes related to abscisic acid synthesis and signaling pathways, 17 genes related to flavonoid biosynthesis, and 15 genes related to carotenoid biosynthesis were positively regulated by H3K9 acetylation [130]. The histone deacetylase 6 (MdHDA6) in apple interacts with the ABA signaling transcription factor ABSCISIC ACID-INSENSITIVE 5 (MdABI5), promoting histone deacetylation to inhibit the expression of downstream genes and reduce drought tolerance [131]. Under CdCl2 stress, Vicia faba histone H3 acetylation at lysine 56 (H3K56ac) is involved in the response to DNA damage during transcription, the S phase, and DNA biosynthesis processes [132]. Cd stress in pakchoi causes the accumulation of ROS and chromatin decondensation. Adding a low concentration of six epi-modification inhibitors (5-AC, RG108, TSA, CUDC101, AT13148, and H89) increases the level of histone acetylation modification and effectively weakens the cell cycle arrest and DNA damage caused by the ROS accumulation induced by Cd [133].

In addition to the well-documented roles of histone lysine methylation and acetylation in regulating stress-responsive gene expression, other residue modifications also play significant roles in plant abiotic stress responses. Histone arginine methylation is usually catalyzed by protein arginine methyltransferases (PRMTs) and occurs on the arginine residues at histone H3 (positions 2, 8, 17, and 26) and histone H4 (position 3). Overexpression of maize protein arginine methyltransferase (ZmPRMT1) by *Arabidopsis thaliana* can enhance its heat tolerance and promote earlier flowering [134].

Histone phosphorylation, which primarily occurs at serine, threonine, and tyrosine residues, demonstrates dynamic changes in plants under environmental stresses such as salt and low-temperature stress [135]. Genome-wide ChIP-Seq analysis revealed that under the treatment of PEG, the kinases MUT9-LIKE KINASE 1 (MLK1) and MUT9-LIKE KINASE 2 (MLK2) promote increased phosphorylated histone H3 threonine 3 (H3T3ph) modification levels in the repetitive Knob region, participating in chromatin structural remodeling to cope with osmotic stress [135].

Histone ubiquitination mainly occurs in histones H2A and H2B. Existing studies have shown that histone H2B monoubiquitination (H2Bub1) plays an important regulatory role in the process of plants responding to salt stress. H2Bub1 enhances the salt tolerance ability of Arabidopsis thaliana by participating in the regulation of salt stress-induced microtubule depolymerization and the PROTEINTYROSINE PHOSPHATASE1-MAP KINASE PHOSPHATASE3/6 (PTP-MPK3/6) signaling pathway [136]. Other research shows that the E2 conjugating enzymes UBIQUITIN CARRIER PROTEINs (UBCs) -1 and -2 in Arabidopsis thaliana mediate the ubiquitination of H2B. Under salt stress, the H2Bub1 mediated by ubc1/2 enhances the level of H3K4me3 in MYB42 and MPK4, while MYB42 directly binds to the promoter of salt overly sensitive 2 (SOS 2) and mediates its rapidly induced expression after salt treatment [137]. The latest study on rice by Ma et al. [138] found that drought stress can lead to an increased H2Bub1 level of the drought tolerance factor OsbZIP46 and its target gene RAB21

### 3.3. Non-Coding RNA Adaptation to Climate Change

Several conserved miRNAs, such as miR169, miR408, miR396, miR171, miR164, miR398, miR399 and miR2118, play crucial roles in response to drought stress in *Oryza sativa* [139]. A comprehensive study elucidating the complex regulatory network of drought response in maize (*Zea mays*) revealed that multiple miRNAs (zma-miR167e, zma-miR167j, zma-miR167f, miR5072, zma-miR529, miR397 and miR6214) participate in drought resistance by targeting various genes, encoding such as SQUAMOSA promoter binding proteins (SBP transcription factors), laccases, Auxin Response Factors (ARFs), MYB transcription factors, and antioxidant enzymes [140]. The miR156/SQUAMOSA PROMOTER BINDING PROTEIN-LIKE13 (miR156/SPL13) module regulates salt stress by inducing the expression of MdWRKY100 in apple [141]. Furthermore, studies in *Populus trichocarpa* demonstrated that DNA methylation under temperature stress conditions can modulate the expression of miRNA genes, thereby indirectly affecting the expression of their target genes [142].

In recent years, an increasing number of studies have shown that lncRNAs are related to plants’ responses to environmental changes. *FLOWERING LOCUS C* (*FLC*) is a key factor in the flowering pathway of *Arabidopsis thaliana* COOLAIR and COLDAIR, two lncRNAs near *FLC*; after receiving cold signals, the COOLAIR promoter induces COOLAIR expression, which targets *FLC* for degradation, while COLDAIR is located in *FLC’s* intron region and can directly recruit PRC2 to mediate methylation, thereby silencing *FLC* [143]. *Arabidopsis* lncRNA *AUXIN-REGULATED PROMOTER LOOP*(*APOLO*) directly regulates the 3D chromatin conformation of the RHD6 locus (H3K27me3 deposition and R-loop site changes) by interacting with WRKY42, thereby activating RHD6 and promoting root hair cell division when under cold stress [144]. The miR394-regulated *LEAF CURLING RESPONSIVENESS* (*ZmLCR*) gene participates in maize drought tolerance [145]. In *Arabidopsis*, WGCNA analysis of the wild type and PV-NES and NLS-PV mutants under salt stress found that under hypertonic and salt stress, lncRNAs regulate the genes related to cell wall structure, plasma membrane components, and osmotic substances through trans-acting [146]. In another report, the overexpression of soybean lncRNA77580 enhanced abiotic stress tolerance compared to the wild type [147]. Under drought stress, the expression of Sly-miR169c is downregulated, while its target lncRNA535 is upregulated. These lncRNAs function as transcriptional regulators of drought-responsive genes, participating in the modulation of various pathways in tomato, including stress tolerance, signal transduction, and transport activities [148]. In cotton, the overexpression of the salt stress-induced lncRNA-973 improves the tolerance to salt stress [149]. Chen et al. detected 69 upregulated and 75 downregulated lncRNAs in rice under Cd stress. Pathway analysis showed significant changes in phenylpropanoids, phenylalanine metabolism, and photosynthesis-related genes [150]. Under strong light, MdLNC610 can enhance the promoter activity of *1-aminocyclopropane-1-carboxylate oxygenase* (*MdACO1*), promoting the release of ethylene and the accumulation of anthocyanins in apples [151]. SVALKA is a long non-coding cis-natural antisense RNA. SVALKA mediates and regulates the expression of the C-Repeat Dehydration Binding Factor 1 (*CBF1*) transcription factor, regulates the histone methylation of *CBF3*, and finally responds to low-temperature stress [152].

It has been reported that most circular RNAs (circRNAs) regulate the growth, development, and stress responses of plants. For example, under low-temperature treatment, 1759 circRNAs were induced in tomato leaves [153]. In maize, 24 and 22 differentially expressed circRNAs were obtained from leaves and roots, respectively, which responded to nitrogen deficiency stress [154]. Through studies on two different salt-sensitive varieties, we identified 2292 potential full-length circRNA sequences in salt-treated maize seedlings, among which 371 showed a responsiveness to salt treatment [155].

Yao et al. found that in wheat seedlings, cold, heat, salt, or drought stress significantly altered the expression of four natural antisense transcript-derived siRNAs (nat-siRNAs). Among them, nat-siRNA005047_0654_1904.1 was significantly upregulated under low-temperature stress but downregulated under other abiotic stresses [156]. In *Arabidopsis thaliana*, TAS1 tasiRNAs also target the heat stress transcription factor genes HEAT-INDUCED TAS1 TARGET 1 (HTT1) and HTT2 to regulate plant thermotolerance [157]. The maize *ZmTAS3j*-overexpressed lines increased the indole-3-acetic acid level in the roots and enhanced their reactive oxygen species scavenging ability through the zma-miR390-ZmTAS3j-tasiARF-ZmARF3-SAURs pathway, thus improving their tolerance to lead [158]. All the examples mentioned in this article are presented in Table 1.

## 4. Epigenetic Stress Memory and Transgenerational Inheritance

During their entire growth phase, plants are exposed to continuously changing climates in nature, particularly adverse extreme climates that can impose varying degrees of stress on their growth and development. As a result, plants have evolved diverse mechanisms to cope with various extreme climate changes. Epigenetic plasticity serves as an effective strategy for plants to respond to environmental stresses caused by climate change. Plants can store short-term stress experiences and react to subsequent stresses—a capability known as somatic stress memory (SSM), which may persist from days to weeks [159]. This type of stress memory, stored at the somatic level and unable to be transmitted to the next generation, is also known as short-term memory. As part of the primary response, environmental stress triggers rapid epigenetic modifications [160]. This initial exposure effectively “primes” the plant by altering their epigenetic landscape, preparing them for future stress events [161]. Stress priming manifests as an enhanced response to subsequent stress encounters, allowing for a more efficient defense activation or resource allocation. This process appears intrinsically linked to the reestablishment of epigenetic modifications after DNA replication. During mitosis in cell division, cells have the opportunity to maintain or reprogram established epigenetic marks. However, with increasing cell divisions and the prolonged absence of stress stimuli above a certain threshold, this epigenetic landscape may become progressively “diluted” [162], even leading to stress memory “forgetting”. This hypothesis also explains why gradual or increasing stress may generate better stress resistance in future encounters compared to sudden exposure to high-level stress [160]. Another mechanism for “forgetting” stress memory is the resetting of stress memory through autophagy in memory-carrying cells [163].

The molecular basis of stress memory involves the complex interplay of specific epigenetic marks. DNA methylation is a key player, with stress-induced methylation patterns persisting after the stress is removed, contributing to this memory. Neves et al. found that in citrus, compared with the first experience of drought stress, the whole-genome methylation patterns of plants that suffered three drought cycles were altered, leading to an increase in the level of abscisic acid. [164]. Similarly, histone modifications, such as altered histone acetylation at defense gene promoters and persistent H3K4me3 marks, as well as histone variations and chaperones, are implicated in maintaining stress memory. In cotton, H3K4me3 is essential for the upregulation of memory genes *9-cis-epoxycarotenoid dioxygenase* (*GhNCED9*), *Pyrabactin resistance 1-like* (*GhPYL9-11A*), *delta1-pyrroline-5-carboxylate synthase 1* (*GhP5CS1*), and *sucrose non-fermenting-1-related protein kinase* (*GhSnRK2*) during repeated drought stress [165]. Non-coding RNAs also contribute to epigenetic memory by mediating the stable transmission of environmentally induced information and maintaining altered expression levels of stress-responsive genes after the cessation of stress, enhancing priming for future stress. A total of 198 differentially expressed 21 nt siRNAs were identified in *Coffea canephora* L. under different cycles of drought treatments, and the majority of their target genes were transcription factors [166]. In durum wheat, Liu et al. discovered the differences in the expression of microRNAs (miRNAs) after repeated drought events, and their target sites play a crucial molecular role in stress adaptation [167]. The correlation analysis between lncRNAs and related mRNAs revealed three mRNA transcripts (tcon_00028567, OS02T0626200-01, and OS04T0412225-00) involved in different pathways and associated with short-term drought memory in rice [168]. In switchgrass, the levels of lncRNAs targeting the biosynthesis of abscisic acid and trehalose increased during the first and second drought cycles, while the lncRNAs regulating ethylene signaling were inhibited during the second drought cycle, thus preventing leaf senescence and supporting plant development [169].

Beyond the individual, a more complex phenomenon is transgenerational epigenetic inheritance (TEI), where stress-induced epigenetic changes are transmitted to subsequent generations [170,171], also called long-term memory. This is distinct from somatic memory, which is typically maintained within the individual through mitotic divisions but erased later in the life cycle or in the offspring to ensure normal development and adaptation to seasonal change. For TEI to occur, epigenetic marks or their effects must be transmitted through the germline via meiosis. It remains unclear how plants overcome epigenetic modification reprogramming events during meiosis to transmit stress memories. Current studies suggest that the transgenerational inheritance of stress memories may be associated with the RdDM pathway [172], and plant genomic imprinting [173,174,175]. Small interfering RNAs (siRNAs) silence transposons through the RNA-dependent DNA methylation (RdDM) pathway, ensuring the regulation of transposons in plant germ cells and the maintenance of genomic imprinting [176].

Evidence for stress-induced transgenerational epigenetic changes and the formation of heritable epialleles is accumulating. Studies have demonstrated a link between inherited epigenetic variations and adaptive phenotypes in offspring, particularly in response to stress. Multi-generational stress exposure, as shown with drought tolerance in rice, can lead to heritable epimutations [177]. Under heat treatment, the loop formed by RELATIVE OF EARLY FLOWERING 6 (REF6) and HEAT SHOCK TRANSCRIPTION FACTOR A2 (HSFA2) reduces the aggregation of tasiRNA, thereby upregulating the expression of HTT5. This leads to early flowering and decreased immunity in *Arabidopsis thaliana*, and these traits can be inherited by the offspring [178]. In apomictic dandelion lineages, changes in the overall proportion of 21 nt and 24 nt sRNAs reveal the memory of offspring to drought and salicylic acid treatment two generations ago [179].

Despite the progress, numerous complexities and open questions surround stress memory and TEI. The duration of epigenetic memories, whether within an individual or across generations, remains an active area of investigation. The mechanisms of memory erasure and resetting, potentially involving enzymes like DNA demethylases and histone deacetylases, are not fully understood. It is also unclear whether stress memory dynamics follow a gradual accumulation or operate via a threshold mechanism, and how plants precisely sense and measure the duration and intensity of stress [160].

## 5. Conclusions

Plants’ capacity to adapt to climate change will profoundly impact species’ survival and extinction, agricultural and environmental sustainability, and food security. Unlike animals that can actively migrate to adapt to different environments, plants can only passively respond to environmental changes. Numerous studies have shown that plants can perceive and respond to climate change through epigenetic mechanisms, striving to maintain their growth, development, and reproduction in complex and variable environments. Unlike permanent genetic mutations, this flexible and dynamic gene expression regulation enables plants to rapidly adjust their responses to environmental fluctuations without altering the underlying DNA sequence. The core epigenetic mechanisms involved include a complex network of DNA methylation, histone modifications, and non-coding RNA. However, despite the enormous potential of plant epigenetics in adapting to climate change, our understanding remain highly limitated. Currently, knowledge of plant epigenetic regulatory networks under complex environmental stresses is still shallow, and the interactions between different epigenetic mechanisms and other physiological processes require further investigation. Moreover, most epigenetic modifications may be reset during generational transmission. Deciphering the transgenerational inheritance mechanisms of epigenetics and applying them to screen for stable and heritable epigenetic variations are key challenges in translating epigenetic research into improving plant stress resistance.

Looking ahead, with the continuous development and refinement of technologies such as high-throughput sequencing, single-cell epigenomic sequencing, and epigenetic editing, we are poised to gain deeper insights into the epigenetic regulatory networks of plants under climate change and fully reveal the molecular mechanisms of plants’ adaptation to climate change. This will provide a theoretical foundation and technical support for breeding new plant varieties adapted to climate change, such as developing efficient, precise, and targeted epigenetic breeding technologies, including the site-specific editing of DNA methylation, RNA methylation, and histone modifications to precisely regulate plant epigenetic marks and enhance stress resistance. At the same time, integrating multi-omics technologies to comprehensively analyze plant responses to climate change at the genomic, transcriptomic, proteomic, and metabolomic levels will help construct a more robust theoretical framework for plant adaptation to climate change.

## Figures and Tables

**Table 1:** Reported studies of epigenetic responses to climate change in recent years.

Species	Epigenetic Modifications	Stress	Key Proteins Involved	Reference
*Arabidopsis thaliana*	CG methylation	Cd		[77]
*Arabidopsis thaliana*	6mA	Col stress		[96]
*Arabidopsis thaliana*	H3K4me3	drought	OST1	[100]
*Arabidopsis thaliana*	H3K4me3	osmotic stress	AtMYB44, ABI1, ABI2, HAI1	[101,102]
*Arabidopsis thaliana*	H3K9me2	salt stress	salt-responsive genes	[111]
*Arabidopsis thaliana*	H3 acetylation	cold stress	ARR1	[116]
*Arabidopsis thaliana*	H4K5ac	heat stress	SOM	[118]
*Arabidopsis thaliana*	Histone hyperacetylation	heat stress	HD2B, HD2C	[121]
*Arabidopsis thaliana*	H3K9ac, H3K14ac	heat stress	HSFA3, UVH6	[122]
*Arabidopsis thaliana*	H3K56ac	heat stress	HsfA2, Hsa32	[123]
*Arabidopsis thaliana*	H3K9ac, H3K14ac	high salt stress	CTL-1, PGX3/MYB54	[125]
*Arabidopsis thaliana*	H3T3ph	osmotic stress	Knob region	[135]
*Arabidopsis thaliana*	H2Bub1	salt stress		[136]
*Arabidopsis thaliana*	H2Bub1	salt stress	MYB42, MPK4	[137]
*Arabidopsis thaliana*	COOLAIR, COLDAIR	low-temperature stress	FLC	[143]
*Arabidopsis thaliana*	APOLO	cold stress	RHD6	[144]
*Arabidopsis thaliana*	SVALKA	low-temperature stress	CBF1	[152]
*Arabidopsis thaliana*	TAS1 tasiRNAs	heat stress	HTT1, HTT2	[157]
*Beta vulgaris*	H3K9ac, H3K27ac	salt stress	POX	[127]
*Beta vulgaris* L.	DNA methylation	salt stress		[85]
*Betula platyphylla* Suk.	DNA methylation	heat stress	BpNST1/2, BpSND1	[92]
*Brassica napus*	DNA methylation	PEG	BnP5CSA	[70]
*Citrus*	DNA methylation	salt stress	ACO1	[87]
*Cucumis sativus*	DNA methylation	low-temperature stress		[90]
*Glycine max*	DNA methylation	salt stress		[83]
*Glycine max*	H3K4me3/H3K4me2	salt stress		[83]
*Glycine max*	lncRNA77580	drought		[147]
*Gossypium hirsutum*	lncRNA-973	salt stress		[149]
*Hibiscus cannabinus* L.	Cd	Cd	NPF2.7	[76]
*Hordeum vulgare*	DNA methylation	water stress		[66]
*Hordeum vulgare*	DNA demrthylation	heat stress		[94]
*Hordeum vulgare*	DNA methylation	mild low-temperature stress		[94]
*Hordeum vulgare*	H3K9ac, H3K4me3	heat stress		[94]
*Lemna minor*	CG and CHG methylation	heat stress		[93]
*Malus pumila* Mill	Histone deacetylation	drought		[131]
*Malus pumila* Mill	miR156, SPL13	salt stress	MdWRKY100	[141]
*Malus pumila* Mill	MdLNC610	strong light		[151]
*Medicago ruthenica*	DNA methylation	drought		[68]
*Medicago sativa*	DNA methylation	drought		[69]
*Medicago sativa*	DNA methylation	salt stress		[84]
*Oryza sativa*	DNA methylation	drought		[63]
*Oryza sativa*	DNA methylation	Hg	mercury resistance-related genes	[79]
*Oryza sativa*	DNA methylation	Cd	genes related to the plant cell wall and oxidative stress	[80]
*Oryza sativa*	CG methylation	low-temperature stress	NLR	[91]
*Oryza sativa*	6mA	heat stress		[99]
*Oryza sativa*	6mA	salt stress		[99]
*Oryza sativa*	6mA	cold stress		[96]
*Oryza sativa*	H3K36me3	drought	MYB48-1	[104]
*Oryza sativa*	H3K4me3/H3K27me3	drought	dehydrin gene cluster	[105]
*Oryza sativa*	H3K34me3	Saline–alkali stress	OsHKT1;5	[109]
*Oryza sativa*	H3K27me3	high salt stress	OsMYB91	[110]
*Oryza sativa*	H3K27me3	heat stress	OsMADS82, OsMADS87, AGL36	[112]
*Oryza sativa*	deacetylase	cold stress	OsbZIP46	[117]
*Oryza sativa*	H4K5 deacetylation, H4K8 deacetylation	salt stress	OsPP2C49	[128]
*Oryza sativa*	H2Bub1	drought	OsbZIP46, RAB21	[138]
*Pakchoi*	Histone acetylation	Cd		[133]
*Phytolacca americana*	DNA methylation	Mn/Cd		[75]
*Populus tomentosa*	non-CG methylation	drought		[65]
*Rosa chinensis*	H3K27me3	low-temperature stress	RcAG	[114]
*Sea buckthorn*	H3K9ac	drought		[130]
*Solanum lycopersicum*	CG methylation	Fe	SlPME53	[74]
*Solanum lycopersicum*	DNA methylation	salt stress	SlDML1, SlDML3, SlDML4, SlHKT1, SlNHX1, SlSOS1	[89]
*Solanum lycopersicum*	H3K9me2	drought	Asr2	[106]
*Solanum lycopersicum*	H3K27me3	drought	Asr1	[107]
*Solanum lycopersicum*	lncRNA535	drought		[148]
*Triticum aestivum*	DNA methylation	osmotic stress and salt stress	TaGAPC1	[67]
*Triticum aestivum*	CG methylation	Pb/Cd/Zn	metal detoxification transporters	[78]
*Triticum aestivum*	H3K36me3	cold stress	VRN1	[113]
*Triticum aestivum*	nat-siRNA005047_0654_1904.1	low-temperature stress		[156]
*Vicia faba*	H3K56ac	Cd		[132]
*Zataria multiflora*	DNA methylation	Cd		[82]
*Zea mays*	DNA methylation	water stress		[64]
*Zea mays*	H3K9ac	heat stress	ZmHsf-01, ZmHsf-15	[119]
*Zea mays*	H3K9ac/H3K5ac	high salt stress		[126]
*Zea mays*	zma-mir167e, zma- miR167j, zma-mir167f, miR5072, zma-mir529, miR397, miR6214	drought		[140]
*Zea mays*	tasiARF	Pb		[158]
*Zea mays*	CHH methylation	salt stress		[86]
